# Prospective relations of maternal reward-related eating, pregnancy ultra-processed food intake and weight indicators, and feeding mode with infant appetitive traits

**DOI:** 10.1186/s12966-022-01334-9

**Published:** 2022-08-03

**Authors:** Jenna R. Cummings, Myles S. Faith, Leah M. Lipsky, Aiyi Liu, Jan T. Mooney, Tonja R. Nansel

**Affiliations:** 1 Social and Behavioral Sciences Branch, Division of Population Health Research, Eunice Kennedy Shriver National Institute of Child Health and Human Development, 6710B Rockledge Drive, Bethesda, MD 20817 USA; 2 Department of Counseling, School, and Educational Psychology, Graduate School of Education, University at Buffalo – SUNY, 420 Bady Hall, Buffalo, NY 14250 USA; 3 Biostatistics and Bioinformatics Branch, Division of Population Health Research, Eunice Kennedy Shriver National Institute of Child Health and Human Development, 6710B Rockledge Drive, Bethesda, MD 20817 USA; 4 Health Psychology PhD Program, University of North Carolina at Charlotte, Colvard Hall, 9201 University City Boulevard, Charlotte, NC 28223 USA

**Keywords:** Infant Appetitive Traits, Pregnancy, Reward-Related Eating, Diet, Weight, Breastfeeding

## Abstract

**Background:**

Infant appetitive traits including eating rate, satiety responsiveness, food responsiveness, and enjoyment of food predict weight gain in infancy and early childhood. Although studies show a strong genetic influence on infant appetitive traits, the association of parent and infant appetite is understudied. Furthermore, little research examines the influence of maternal pregnancy dietary intake, weight indicators, and feeding mode on infant appetite. The present study investigated relations of maternal reward-related eating, pregnancy ultra-processed food intake and weight indicators, and feeding mode with infant appetitive traits.

**Methods:**

Mothers in the Pregnancy Eating Attributes Study (458 mothers enrolled, 367 retained through delivery) completed self-report measures of reward-related eating, and principal component analysis yielded two components: (1) food preoccupation and responsiveness and (2) reinforcing value of food. Mothers completed 24-h dietary recalls across pregnancy, and the standardized NOVA (not an acronym) system categorized recalled foods based on processing level. Maternal anthropometrics were measured across pregnancy. At infant age 6 months, mothers reported on feeding mode and infant appetitive traits. Linear regressions were conducted predicting infant appetitive traits from household income-poverty ratio (step 1); maternal reward-related eating components (step 2); pregnancy ultra-processed food intake (% of energy intake), early pregnancy body mass index, and gestational weight gain (step 3); and exclusive breastfeeding duration (step 4).

**Results:**

A 1-SD greater maternal food preoccupation and responsiveness was associated with 0.20-SD greater infant satiety responsiveness (*p* = .005). A 1-SD greater % energy intake from ultra-processed foods during pregnancy was associated with 0.16-SD lower infant satiety responsiveness (*p* = .031). A 1-SD longer exclusive breastfeeding duration was associated with 0.18-SD less infant food responsiveness (*p* = .014). Other associations of maternal reward-related eating, pregnancy ultra-processed food intake and weight indicators, and feeding mode with infant appetitive traits were non-significant.

**Conclusions:**

Proximal early-life environmental factors including maternal pregnancy dietary intake and feeding mode may facilitate or protect against obesogenic infant appetitive traits, whereas infant appetite may not parallel maternal reward-related eating. Further investigation into the etiology of appetitive traits early in development, particularly during solid food introduction, may elucidate additional modifiable risk factors for child obesity.

**Trial registration:**

Clinicaltrials.gov. Registration ID – NCT02217462. Date of registration – August 13, 2014.

**Supplementary Information:**

The online version contains supplementary material available at 10.1186/s12966-022-01334-9.

## Background

The behavioral susceptibility theory of obesity posits that appetitive traits—enduring predispositions towards food including eating rate, satiety responsiveness, food responsiveness, and enjoyment of food—influence susceptibility to weight gain in an obesogenic environment [1–4]. Longitudinal studies indicate that faster eating rates [5–10], lower satiety responsiveness [5, 6, 11], greater food responsiveness [5, 6, 11], and greater enjoyment of food [6] predict weight gain as early as infancy. Given the high prevalence of obesity in children [12] and its impact on multiple health outcomes [13], understanding the etiology of infant appetitive traits is an important area of investigation.

Although multiple studies have shown a sizable genetic influence on infant appetitive traits [14–17], the association of parent and infant appetite is understudied. Reward-related eating is the propensity to consume food for its rewarding characteristics [18] and conceptually overlaps with appetitive traits [19]. Dimensions of reward-related eating include loss of control eating, lack of satiety, preoccupation with food, and greater reinforcing value of food [18, 20]. Other than one study indicating that greater maternal loss of control eating was associated with greater infant food responsiveness [21], no studies to our knowledge have examined the relations of parent reward-related eating with infant appetitive traits.

In addition to parent reward-related eating, maternal dietary intake and weight during pregnancy may influence infant appetitive traits through the prenatal environment [15]. Ultra-processed foods (typically containing sugar, oil, salt, and other additives) [22] stimulate neural reward circuitry and increase appetitive drive in adults [23], suggesting that maternal intake of these foods during pregnancy may influence infant appetitive traits. Experimental research with rodents demonstrated that a maternal “junk food” diet (i.e., biscuits, marshmallows, cheese, jam doughnuts, chocolate chip muffins, butter flapjacks, potato crisps, and caramel/chocolate bars) and diet-induced obesity during pregnancy caused amplified preferences for junk food, hyperphagia, and propensity for obesity in offspring [24, 25]. However, in humans, only one study to our knowledge has examined the relation of maternal pregnancy dietary intake with infant appetitive traits [26], and the relations of maternal early pregnancy body mass index (BMI) and gestational weight gain (GWG) with infant appetitive traits have not been investigated.

Feeding mode during infancy may further influence appetitive traits by affecting the early life environment [15]. Infants may learn to self-regulate their intake better during breastfeeding than bottle feeding due to greater control over feed size [27] and higher required effort for feeding [28]. One study found that a longer duration of breastfeeding predicted greater slowness in eating but no other appetitive traits [29], while another study found that longer breastfeeding duration predicted greater satiety responsiveness but relations with other infant appetitive traits were unexamined [30].

The present study addressed knowledge gaps by examining relations of maternal reward-related eating, pregnancy ultra-processed food intake and weight indicators, and feeding mode with infant appetitive traits at 6 months of age. We hypothesized that greater maternal reward-related eating and greater pregnancy ultra-processed food intake, BMI, and GWG, as well as shorter exclusive breastfeeding, would predict faster eating rates, lower satiety responsiveness, greater food responsiveness, and greater enjoyment of food in infants. Additionally, given the dearth of evidence regarding associations of sociodemographic characteristics with infant appetitive traits, we tested for these associations without specific hypotheses.

## Methods

### Design, setting, participants, and procedures

The Pregnancy Eating Attributes Study (PEAS) examined the influence of reward-related eating on diet quality and weight during pregnancy and postpartum in women receiving prenatal care in Chapel Hill, North Carolina, United States [31]. Potential participants were identified through the electronic medical records database and were provided with information regarding study participation by research staff. Eligibility criteria were previously described in detail [31] and included BMI ≥ 18.5 kg/m^2^ and absence of pre-existing diabetes, any medical condition contraindicating study participation, participant-reported eating disorder, and medication use that could affect dietary intake or weight.

Mothers provided informed consent and, at six visits, completed behavioral measures through a secure online data collection system. Certified researchers assessed mothers’ anthropometrics. Visits occurred at < 12 weeks (baseline/first trimester), 16–22 weeks (second trimester), and 28–32 weeks (third trimester) gestation and at 4–14 weeks (~ 2 months), 23–31 weeks (~ 6 months), and 50–58 weeks (~ 1 year) postpartum. Procedures were approved by the University of North Carolina Institutional Review Board (study #18–2030) and were in accordance with the ethical standards of the Helsinki Declaration of 1975 as revised in 1983. Recruitment was initiated in November 2014 and ended in December 2016, and data collection completed in June 2018. See Fig. 1 in Nansel et al. (2020) for a flow diagram of the number of mothers at each study stage [32]. Of 458 mothers enrolled, 367 and 321 were retained through delivery and one-year postpartum, respectively [33]. The present study was a secondary data analysis using all available data for variables of interest.

### Measures

#### Dependent variables

##### Infant appetitive traits

At 6 months postpartum, mothers completed the 17-item Baby Eating Behavior Questionnaire (BEBQ) [34]. The BEBQ assesses appetitive traits including slowness in eating (e.g., “My baby fed slowly”; 4 items; *α* = 0.62), satiety responsiveness (e.g., “My baby got full before taking all the milk I thought s/he should have”; 3 items; *α* = 0.39), food responsiveness (e.g., “If given the chance, my baby would always be feeding”; 6 items; *α* = 0.80), and enjoyment of food (e.g., “My baby loved milk”; 4 items; *α* = 0.75) in infants during the period of milk feeding. Mothers rated items on a 5-point Likert scale (1 = “Never,” 5 = “Always”). Items from each subscale were averaged, with higher scores indicating slower eating rates, greater satiety responsiveness, greater food responsiveness, and greater enjoyment of food.

#### Independent variables

##### Maternal reward-related eating

Given the absence of a gold-standard self-report measure of reward-related eating, mothers completed four measures assessing dimensions of reward-related eating at baseline: the modified Yale Food Addiction Scale (mYFAS) [35], the Power of Food Scale (PFS) [36], the Multiple Choice Procedure (MCP) [37], and the Reinforcing Value of Food Questionnaire (RVFQ) [38]. The 9-item mYFAS assesses addictive-like responses (e.g., excessive intake, persistent desire) to highly palatable foods based on the Diagnostic and Statistical Manual of Mental Disorders (4^th^ ed.) criteria for substance dependence [35]. Mothers rated items on an 8-point Likert scale from 1 (“Never”) to 8 (“Every day”). Responses to items were dichotomized (0 or 1) based on published cut-off values [35] and summed (*α* = 0.71), with higher sums indicating greater addictive-like eating.

The 15-item PFS measures motivation to consume highly palatable foods in response to environmental cues [36]. On a 5-point Likert scale from 1 (“Do not agree”) to 5 (“Strongly agree”), mothers rated items from three subscales regarding motivations for food available but not physically present (e.g., “I find myself thinking about food even when I am not physically hungry”), food physically present but not tasted (e.g., “If I see or smell a food I like, I get a powerful urge to have some”), and food tasted but not consumed (e.g., “Just before I taste a favorite food, I feel intense anticipation”); the mean of these subscales was calculated (*α* = 0.92), with higher values indicating greater motivation to consume highly palatable foods.

The MCP has previously been used to assess individual differences in the reinforcing value of drugs [37]; PEAS investigators modified this measure to assess individual differences in the reinforcing value of food. Mothers chose between a specified food and increasing amounts of money. The price at which mothers chose the money over the food (breakpoint) indicated their reinforcing value of food, with higher breakpoints indicating greater reinforcing value. The RVFQ, previously adapted from measures assessing reinforcing value of substances, also assesses individual differences in the reinforcing value of food [38]. Mothers reported the number of portions of a specified food they would purchase at varying prices. The RVFQ yields five reinforcing value of food indices: breakpoint (i.e., first price at which number of portions selected was zero), intensity (i.e., number of portions selected at the lowest price), Omax (i.e., product of portions selected and price), Pmax (i.e., price at which expenditure was maximized), and elasticity (i.e., sensitivity of decrease in consumption to increase in price, calculated using the modified exponential demand equation) [39]. Before completing the MCP and the RVFQ, mothers rated their liking of 18 highly palatable foods and the two highest-rated foods became the specified foods used in those questionnaires. During the second and third trimesters of pregnancy, mothers repeated completion of the PFS, MCP, and RVFQ; mean PFS, MCP, and RVFQ scores across baseline and pregnancy were calculated because of robust correlations across repeated scores [33].

##### Maternal ultra-processed food intake during pregnancy

Mothers were asked to complete the well-validated Automated Self-Administered 24-Hour Dietary Recall (ASA24) [40] once within each trimester visit window, indicating all foods consumed in the past 24 h, including details on food preparation, brands, portion size, and additions. The ASA24 program assigned food codes from the U.S. Department of Agriculture Food and Nutrient Database for Dietary Studies (FNDDS) and provided nutrition information including kilocalories [40]. Research staff at the University of North Carolina Nutrition and Obesity Research Core corrected implausible and missing food codes and nutrition information. Records (1.9%) with implausible daily energy intake (< 600 kcal/day), based on established cutoffs adjusted for increased energy requirements of pregnancy, were excluded from analysis resulting in exclusion of one participant [41, 42].

Food codes were categorized according to the standardized NOVA (not an acronym) classification system into (a) unprocessed or minimally processed foods (e.g., fresh and frozen fruits and vegetables), (b) processed culinary ingredients (e.g., oils, vinegars), (c) processed foods (e.g., natural cheese, fruit preserves), and (d) ultra-processed foods (e.g., confectionery desserts, sweetened drinks, ‘instant’ foods, reconstituted meats, sweet or savory packaged snacks) [43]. For food codes indicating a homemade recipe, underlying ingredient codes and correspondent nutrition information were obtained from the FNDDS and categorized according to NOVA [43]. Given that there is little change in dietary intake across pregnancy trimesters [44–46], total daily energy intake during pregnancy was calculated by pooling data across this period. Percent daily energy intake from ultra-processed foods during pregnancy was calculated by dividing the average total daily energy from ultra-processed foods by the average total daily energy intake [43]. Calculating percent daily energy intake from ultra-processed foods may reduce bias introduced by non-differential calorie misreporting from all foods [47].

##### Maternal weight indicators

At baseline, trained researchers measured maternal height using a wall-mounted stadiometer and weight using a standing scale and recorded to the nearest 0.1 cm and 0.1 kg, respectively. Each measure was obtained twice; if the two measurements varied by more than 1 cm (height) or 0.2 kg (weight), a third measure was taken. Early pregnancy BMI was calculated from the mean of the two closest height and weight measurements. Patient medical records indicated maternal weight at each prenatal medical visit. GWG was calculated as the difference between baseline weight and the last prenatal medical visit weight [*M*(*SD*) = 0.35(0.75) weeks prior to delivery]. GWG was categorized as inadequate, adequate, or excessive using 2009 Institute of Medicine guidelines for optimal range of GWG [48]. In rodent models, diet-induced obesity during pregnancy caused appetitive changes in offspring [25], so GWG was dummy coded for analysis [0 (inadequate or adequate), 1 (excessive)].

##### Feeding mode

At each postpartum visit, mothers reported feeding modes including breastfeeding from the breast, breastfeeding from the bottle, formula feeding, and complementary feeding [49]. Exclusive breastfeeding was defined as breastfeeding their infant from the breast or feeding their infant breastmilk from a bottle with no feeding of formula or complementary foods. If they were no longer exclusively breastfeeding, mothers reported at what infant age (in months) they introduced other feeding modes. A continuous score of duration of exclusive breastfeeding through infant age of 6 months was calculated.

##### Sociodemographic characteristics

Maternal age at baseline was abstracted from medical records. Mothers reported their race [dummy coded 0 (minority race) and 1 (non-Hispanic white)], total annual household income, and household composition. Income-poverty ratio was calculated by dividing total annual household income by the US Census Bureau 2016 poverty thresholds, accounting for household size and number of children [50].

### Statistical analysis

Analyses were conducted in SPSS Statistics 28.0.0.0 (IBM Corporation, Armonk, NY) using the pairwise deletion approach to account for missing data. Significance was set at *p* < 0.05. Normality of the data was assessed. The mYFAS, MCP, and RVFQ scores showed high skew and kurtosis and were therefore log-transformed, and the RVFQ elasticity score was reverse scored to be consistent with other reward-related eating scores. For ease of interpretation, all continuous variables were z-scored.

Bivariate correlations between variables (uncorrected for multiple comparisons) were conducted (see Additional File 1) to inform subsequent analyses. Given that associations of maternal age and race with infant appetitive traits were non-significant, these variables were not included in subsequent analyses for parsimony. Small to large significant associations were observed among maternal reward-related eating scores. To reduce the maternal reward-related eating data into aggregate units that could be examined simultaneously in subsequent analyses, a principal components analysis (PCA) was conducted using the baseline mYFAS scores and the mean PFS, MCP, and RVFQ scores. PCA was selected to maximize the amount of the total variance explained in maternal reward-related eating scores [51]. An oblique rotation method (promax) was selected because a correlation among components was expected [52].

To test the present study hypotheses, linear regressions were conducted with each infant appetitive trait as a separate dependent variable. Independent variables were entered as follows, in order of temporal precedence and physiological proximity (least to most) relative to infant appetitive traits: Step 1 included household income-poverty ratio, Step 2 added the PCA-determined maternal reward-related eating components, Step 3 added maternal ultra-processed food intake (% of energy intake) during pregnancy, early pregnancy BMI, and GWG, and Step 4 added exclusive breastfeeding duration. Given the potential bias introduced by missing data, post hoc sensitivity analyses were conducted. Specifically, path modeling with full information maximum likelihood (FIML) estimation, a procedure that allows for retention of the maximum number of valid cases while producing unbiased estimates, was conducted in SAS 9.4 (Cary, NC) using PROC CALIS.

## Results

Univariate statistics for variables of interest are presented in Table 1.

**Table 1:** Univariate statistics for variables of interest

	*n*	*M(SD) or n(%)*
**Sociodemographic Characteristics**		
Maternal Age, years	458	30.46 (4.74)
Maternal Race	392	
Minority Race, including Black, Asian, Hispanic or Latino		128 (32.7%)
Non-Hispanic White		264 (67.3%)
Household Income-Poverty Ratio	364	3.84 (1.97)
**Maternal Reward-Related Eating**		
mYFAS	344	0.50 (0.95)
PFS	377	2.20 (0.67)
MCP breakpoint	350	3.67 (4.58)
RVFQ breakpoint	347	7.75 (32.67)
RVFQ intensity	348	3.95 (4.29)
RVFQ Omax	348	4.89 (16.31)
RVFQ Pmax	348	3.80 (16.08)
RVFQ elasticity	347	0.07 (0.22)
**Maternal Pregnancy UPF Intake and Weight Indicators**		
%Energy Intake from UPF	365	52.58 (15.12)
Early Pregnancy BMI, kg/m^2^	458	27.19 (6.94)
GWG	367	
Inadequate or adequate		194 (52.9%)
Excessive		173 (47.1%)
**Feeding Mode**		
Exclusive Breastfeeding Duration, months	302	1.99 (2.63)
**Infant Appetitive Traits**		
Slowness in Eating	229	2.40 (0.62)
Satiety Responsiveness	229	2.24 (0.59)
Food Responsiveness	229	2.23 (0.65)
Enjoyment of Food	229	4.49 (0.47)

Untransformed data are presented. *mYFAS* Modified Yale Food Addiction Scale, *PFS* Power of Food Scale, *MCP* Multiple Choice Procedure, *RVFQ* Reinforcing Value of Food Questionnaire, *UPF* Ultra-Processed Food, *BMI* Body Mass Index, *GWG* Gestational Weight Gain

The PCA of maternal reward-related eating scores yielded a scree plot with two components above the “break,” in which each had an eigenvalue > 1; one-component, two-component, and three-component solutions were further investigated. Table 2 provides estimates from the final two-component solution, which was selected because no components consisted of less than three scores and there were appropriate magnitudes of loadings [51, 52], although one score (MCP breakpoint) cross-loaded. Based on the dimensions of reward-related eating captured by the measures with the highest loading scores on each component, the two components were labeled maternal “food preoccupation and responsiveness” and “reinforcing value of food.” The two components were modestly correlated (*r* = 0.24).

**Table 2:** Maternal reward-related eating component loadings

	**Component 1 “Food Preoccupation and Responsiveness” Loadings**	**Component 2 “Reinforcing Value of Food” Loadings**
mYFAS^a^	.55	-.11
PFS	.83	-.04
MCP breakpoint^a^	.30	.34
RVFQ breakpoint^a^	-.03	.96
RVFQ intensity^a^	.72	.12
RVFQ Omax^a^	.06	.94
RVFQ Pmax^a^	-.12	.97
RVFQ elasticity^ab^	.01	.61

Variable was ^a^log-transformed and ^b^reverse scored. *mYFAS* Modified Yale Food Addiction Scale, *PFS* Power of Food Scale, *MCP* Multiple Choice Procedure, *RVFQ* Reinforcing Value of Food Questionnaire

Estimates from linear regressions predicting infant slowness in eating and satiety responsiveness, and infant food responsiveness and enjoyment of food, are presented in Tables 3 and 4, respectively. Holding household income-poverty ratio constant, a 1-SD greater maternal food preoccupation and responsiveness was significantly associated with 0.20-SD greater infant satiety responsiveness. Holding household income-poverty ratio and maternal reward-related eating constant, a 1-SD greater % energy intake from ultra-processed foods during pregnancy was associated with 0.16-SD lower infant satiety responsiveness. Holding household income-poverty ratio, maternal reward-related eating, and pregnancy ultra-processed food intake and weight indicators constant, a 1-SD longer exclusive breastfeeding duration was associated with 0.18-SD less infant food responsiveness. All other associations of maternal reward-related eating, pregnancy ultra-processed food intake and weight indicators, and feeding mode with infant appetitive traits were non-significant. The direction, magnitude, and significance of the tested associations were consistent in post hoc sensitivity analysis applying FIML (not shown) except for the association of household income-poverty ratio with infant slowness in eating, which was slightly higher in magnitude and statistically significant [*B(SE)* = 0.15(0.07), *p* = 0.037].

**Table 3:** Associations of infant slowness in eating and satiety responsiveness with income-poverty ratio, maternal reward-related eating, pregnancy ultra-processed food intake and weight indicators, and feeding mode

	**Infant Slowness in Eating**			**Infant Satiety Responsiveness**		
**Maternal Variables**	*B(SE)*	*p*	*ΔR*^*2*^	*B(SE)*	*p*	*ΔR*^*2*^
**Step 1**			.02			.01
Income-poverty Ratio	0.13(0.07)	.054		-0.08(0.07)	.271	
**Step 2**			.00			.04
Food Preoccupation and Responsiveness	-0.01(0.07)	.879		0.20(0.07)	.005	
Reinforcing Value of Food	-0.06(0.07)	.402		-0.12(0.07)	.101	
**Step 3**			.02			.02
%Energy Intake from UPF	-0.09(0.07)	.243		-0.16(0.07)	.031	
Early Pregnancy BMI	0.03(0.08)	.718		-0.06(0.08)	.541	
Excessive GWG^a^	-0.23(0.15)	.118		0.02(0.14)	.892	
**Step 4**			.00			.01
Exclusive Breastfeeding Duration	-0.07(0.07)	.346		-0.10(0.07)	.141	

Linear regressions were conducted with independent variables entered as follows: Step 1 included household income-poverty ratio, Step 2 added maternal food preoccupation and responsiveness and maternal reinforcing value of food, Step 3 added maternal UPF intake (% of energy intake) during pregnancy, early pregnancy BMI, and GWG, and Step 4 added exclusive breastfeeding duration. ^a^Dummy coded (0 = Inadequate or adequate, 1 = Excessive). *UPF* Ultra-Processed Food, *BMI* Body Mass Index, *GWG* Gestational Weight Gain

**Table 4:** Associations of infant food responsiveness and enjoyment of food with income-poverty ratio, maternal reward-related eating, pregnancy ultra-processed food intake and weight indicators, and feeding mode

	**Infant Food Responsiveness**			**Infant Enjoyment of Food**		
**Maternal Variables**	*B(SE)*	*p*	*ΔR*^*2*^	*B(SE)*	*p*	*ΔR*^*2*^
**Step 1**			.00			.01
Income-poverty Ratio	0.02(0.07)	.727		-0.12(0.07)	.094	
**Step 2**			.01			.01
Preoccupation with Food	0.09(0.07)	.237		-0.06(0.07)	.374	
Reinforcing Value of Food	0.02(0.07)	.833		-0.06(0.07)	.416	
**Step 3**			.02			.01
%Energy Intake from UPF	0.06(0.07)	.398		-0.03(0.07)	.692	
Early Pregnancy BMI	0.13(0.08)	.106		0.07(0.08)	.370	
Excessive GWG^a^	-0.08(0.15)	.602		0.14(0.15)	.353	
**Step 4**			.03			.02
Exclusive Breastfeeding Duration	-0.18(0.07)	.014		0.14(0.07)	.055	

Linear regressions were conducted with independent variables entered as follows: Step 1 included household income-poverty ratio, Step 2 added maternal food preoccupation and responsiveness and maternal reinforcing value of food, Step 3 added maternal UPF intake (% of energy intake) during pregnancy, early pregnancy BMI, and GWG, and Step 4 added exclusive breastfeeding duration. ^a^Dummy coded (0 = Inadequate or adequate, 1 = Excessive). *UPF* Ultra-Processed Food, *BMI* Body Mass Index, *GWG* Gestational Weight Gain

## Discussion

In this prospective study of mother–child dyads in the Chapel Hill, North Carolina vicinity, we investigated associations of maternal reward-related eating, pregnancy ultra-processed food intake, weight indicators, and feeding mode with infant appetitive traits (eating rate, satiety responsiveness, food responsiveness, and enjoyment of food). Results indicated that greater maternal pregnancy ultra-processed food intake was associated with lower infant satiety responsiveness, and longer exclusive breastfeeding duration was associated with lower infant food responsiveness. Contrary to what was hypothesized, greater maternal food preoccupation and responsiveness was associated with greater infant satiety responsiveness. All other associations of maternal factors with infant appetitive traits were modest in magnitude and statistically non-significant. Overall, these results suggest that proximal early-life environmental factors including maternal pregnancy dietary intake and feeding mode may influence obesogenic infant appetitive traits, whereas maternal reward-related eating may not parallel infant appetite.

The finding that greater maternal ultra-processed food intake during pregnancy was associated with lower infant satiety responsiveness builds upon experimental findings that, in rodents, maternal pregnancy “junk food” diet (i.e., biscuits, marshmallows, cheese, jam doughnuts, chocolate chip muffins, butter flapjacks, potato crisps, and caramel/chocolate bars) and diet-induced obesity caused a propensity for obesity in offspring [24, 25]. The only prior study in humans did not observe a relation of maternal “junk food” diet (i.e., component score with heavy loadings from sweets, sweet drinks, pizza, hot chips, potato chips, cake, chocolate, pancakes, meat pies, white bread, ice cream, salami, other pasta, alcoholic beverages, jams, international takeaway, low caloric drinks, biscuits, sausages, and cream-based dairy) with infant appetitive traits [26]. Differences between findings of the present versus previous study are likely attributable to differences in the dietary assessment methods and in the independent variable definitions. The present study measured dietary intake using 24-h dietary recalls, which have superior validity and provide less biased estimates of intakes compared to the previously used food frequency questionnaire [53], thus providing greater ability to detect associations between maternal dietary intake and infant appetitive traits. Additionally, the previous study did not measure ultra-processed food intake, but rather quantified maternal intake of “junk foods” as reported in the food frequency questionnaire.

Adding to previous literature showing that longer breastfeeding duration predicted greater infant slowness in eating and satiety responsiveness [29, 30], and a meta-analysis indicating a dose-dependent association between longer breastfeeding and decrease in risk of child overweight [54], longer exclusive breastfeeding duration was associated with lower infant food responsiveness. It is unclear why breastfeeding duration has been associated with different infant appetitive traits across studies; however, associations with lower food responsiveness and greater slowness in eating and satiety responsiveness are consistent with the notion that infants may learn to self-regulate their intake better during breastfeeding than bottle feeding [27, 28]. Breastfeeding may facilitate maternal responsive feeding practices (i.e., recognize and respond to infant hunger and fullness signs), which promote self-regulation [55] and prevent excessive weight gain in infants [56]. As breastmilk is a complex biological fluid with hormones including leptin and ghrelin, which are known to support the early control of satiety in infants [57], breastfeeding from either the breast or bottle may plausibly reduce infant food responsiveness.

In contrast to the association of greater maternal loss of control eating with greater infant food responsiveness previously reported [21], findings herein indicated that greater maternal food preoccupation and responsiveness was associated with greater infant satiety, but not food responsiveness, and there were no associations of maternal reinforcing value of food with infant appetitive traits. The difference in findings between studies may be due to different operationalizations of maternal reward-related eating. In the present study, maternal food preoccupation and responsiveness and maternal reinforcing value of food were aggregated from continuous scores of multiple reward-related eating measures, whereas in the prior study, maternal loss of control eating was dichotomized based on presence of at least one severe overeating episode during pregnancy and may represent a more extreme eating behavior. The sample was larger in the present versus prior study; yet, the degree of reward-related eating was lower than estimates of mYFAS scores in national samples of middle-aged and older women [35]. Estimates of PFS scores were comparable to those in a national sample of young adults [58]. The more recently developed Reward-based Eating Drive scale [18], which was designed to capture a fuller range of all dimensions of reward-related eating, may improve future reward-related eating assessment in non-clinical samples. While reward-related eating conceptually overlaps with appetitive traits [19], assessing the traits of eating rate, satiety responsiveness, food responsiveness, and enjoyment of food in parents with a matched measure [59] may strengthen future investigations into the potential overlap of parent and infant appetite.

Although researchers have suggested that sociodemographic characteristics may influence infant appetitive traits [60], this has not been tested in prior work. Only greater household income-poverty ratio was associated with slower infant eating rates in exploratory analysis for the present study. To our knowledge, this is the first study to report an association between income (or a related metric) and infant eating rate, which is an established and reliable predictor of weight status in infants and young children [5–10]. This finding also is consistent with prior work suggesting that greater access to non-food reinforcers (e.g., musical instruments), which may be more available in families with more financial resources, may reduce general appetitive drive in infants [61]. Larger, more representative samples are needed to fully investigate sociodemographic differences in infant appetitive traits.

The present study findings should be considered in light of study strengths and weaknesses. Internal validity was strengthened by the repeated measures of maternal pregnancy dietary intake using 24-h dietary recalls, the least biased self-report available [53]; by directly measuring maternal pregnancy weight indicators; by adequate representation of women with high early pregnancy BMI and excessive GWG; and by the simultaneous testing of multiple potential influences on infant appetitive traits. Although the observational study design limits causal inferences, there was temporal precedence in measurement of sociodemographic characteristics, maternal reward-related eating, pregnancy ultra-processed food intake and weight indicators, and feeding mode relative to infant appetitive traits. Maternal reward-related eating was estimated from scores on four self-report measures assessing different dimensions of reward-related eating, and future research would benefit from determination of a gold-standard measure of this construct. The existing measure of infant appetitive traits is a maternal report, which may introduce bias from mothers’ interpretation of their infants’ appetites and may inflate associations with the other constructs assessed by maternal-report. The construct of infant satiety responsiveness may not have been reliably captured in the present study sample as indicated by low internal consistency among the three items assessing the trait. Sociodemographic characteristics were fairly homogenous in the present study sample with regards to maternal race and household income (consistent with those from Chapel Hill, North Carolina), limiting generalizability to more heterogeneous groups.

## Conclusions

Proximal early-life environmental factors including maternal pregnancy dietary intake and feeding mode may influence infant appetitive traits, particularly satiety and food responsiveness, whereas infant appetite may not parallel maternal reward-related eating. Randomized clinical trials are needed to test whether reducing maternal pregnancy ultra-processed food intake and increasing exclusive breastfeeding duration facilitates infant appetitive traits that lower the risk of excessive weight gain in infancy and early childhood. The role of environmental factors in appetite may intensify later in infancy and in childhood because of the introduction of solid foods [62] and subsequent changes to parental feeding practices [63–65]. Concordance between maternal and child reward-related eating may emerge when children can model maternal eating behaviors with solid food at shared eating occasions. Further investigation into the etiology of appetitive traits early in development may elucidate additional modifiable risk factors for child obesity.

## Supplementary Information

**Additional file 1.** Bivariate Correlations Between Variables of Interest.

## Data Availability

Data described in the manuscript, code book, and analytic code will be made available from the corresponding author upon request pending application and approval.

## Abbreviations

ASA24: Administered 24-Hour Dietary Recall
BEBQ: Baby Eating Behavior Questionnaire
BMI: Body Mass Index
FIML: Full Information Maximum Likelihood
FNDDS: Food and Nutrient Database for Dietary Studies
GWG: Gestational Weight Gain
MCP: Multiple Choice Procedure
mYFAS: Modified Yale Food Addiction Scale
PCA: Principal Components Analysis
PEAS: Pregnancy Eating Attributes Study
PFS: Power of Food Scale
RVFQ: Reinforcing Value of Food Questionnaire
