# Supplementary material for: Prospective relations of maternal reward-related eating, pregnancy ultra-processed food intake and weight indicators, and feeding mode with infant appetitive traits
Source: Int J Behav Nutr Phys Act. 2022 Aug 3;19:100. doi: 10.1186/s12966-022-01334-9 (PMC9351142; doi:10.1186/s12966-022-01334-9)
Supplement: Supplementary file 1 — Additional file 1. Bivariate Correlations Between Variables of Interest. [file 12966_2022_1334_MOESM1_ESM.docx]

Additional File 1

*Bivariate Correlations Between Variables of Interest*

|  | 1. | 2. | 3. | 4. | 5. | 6. | 7. | 8. | 9. | 10. | 11. | 12. | 13. | 14. | 15. | 16. | 17. | 18. |
| --- | --- | --- | --- | --- | --- | --- | --- | --- | --- | --- | --- | --- | --- | --- | --- | --- | --- | --- |
| **Maternal Sociodemographic Characteristics** |  |  |  |  |  |  |  |  |  |  |  |  |  |  |  |  |  |  |
| 1. Age | -- | -- | -- | -- | -- | -- | -- | -- | -- | -- | -- | -- | -- | -- | -- | -- | -- | -- |
| 2. Race^a^ | .20*** | -- | -- | -- | -- | -- | -- | -- | -- | -- | -- | -- | -- | -- | -- | -- | -- | -- |
| 3. Income-Poverty Ratio | .34*** | .34*** | -- | -- | -- | -- | -- | -- | -- | -- | -- | -- | -- | -- | -- | -- | -- | -- |
| **Maternal Reward-Related Eating** |  |  |  |  |  |  |  |  |  |  |  |  |  |  |  |  |  |  |
| 4. mYFAS^b^ | -.14** | -.20*** | -.21*** | -- | -- | -- | -- | -- | -- | -- | -- | -- | -- | -- | -- | -- | -- | -- |
| 5. PFS | -.09 | -.02 | .06 | .29*** | -- | -- | -- | -- | -- | -- | -- | -- | -- | -- | -- | -- | -- | -- |
| 6. MCP Breakpoint^b^ | -.04 | .11* | .14* | -.10 | .26*** | -- | -- | -- | -- | -- | -- | -- | -- | -- | -- | -- | -- | -- |
| 7. RVFQ Breakpoint^b^ | -.02 | -.01 | .09 | .07 | .14* | .30*** | -- | -- | -- | -- | -- | -- | -- | -- | -- | -- | -- | -- |
| 8. RVFQ Intensity^b^ | -.04 | .03 | -.03 | .12* | .41*** | .26*** | .28*** | -- | -- | -- | -- | -- | -- | -- | -- | -- | -- | -- |
| 9. RVFQ Omax^b^ | -.03 | .00 | .09 | .07 | .18*** | .32*** | .94*** | .41*** | -- | -- | -- | -- | -- | -- | -- | -- | -- | -- |
| 10. RVFQ Pmax^b^ | -.04 | -.03 | .11* | .06 | .13* | .28*** | .92*** | .14** | .88*** | -- | -- | -- | -- | -- | -- | -- | -- | -- |
| 11. RVFQ Elasticity^b,c^ | .00 | .02 | .11 | -.03 | .16** | .19*** | .39*** | .17** | .44*** | .49*** | -- | -- | -- | -- | -- | -- | -- | -- |
| **Maternal Pregnancy UPF Intake and Weight Indicators** | | |  |  |  |  |  |  |  |  |  |  |  |  |  |  |  |  |
| 12. Pregnancy %Energy Intake from UPF | -.22*** | .00 | -.15** | .12* | .14** | .15** | .02 | .22*** | .09 | -.03 | -.08 | -- | -- | -- | -- | -- | -- | -- |
| 13. Early Pregnancy BMI | -.03 | -.29*** | -.31*** | .33*** | .00 | -.07 | -.08 | .17** | -.06 | -.12* | -.21*** | .14** | -- | -- | -- | -- | -- | -- |
| 14. Maternal Excessive GWG^d^ | .01 | -.02 | .07 | -.02 | .08 | .09 | .05 | .14* | .08 | .05 | .08 | .15** | .16** | -- | -- | -- | -- | -- |
| **Feeding Mode** |  |  |  |  |  |  |  |  |  |  |  |  |  |  |  |  |  |  |
| 15. Exclusive Breastfeeding Duration | .10 | .20*** | .11 | -.21*** | -.05 | .07 | -.01 | -.06 | -.04 | .01 | .08 | -.06 | -.24*** | .01 | -- | -- | -- | -- |
| **Infant Appetitive Traits** |  |  |  |  |  |  |  |  |  |  |  |  |  |  |  |  |  |  |
| 16. Slowness in Eating | .03 | .07 | .13* | -.11 | .02 | -.05 | -.06 | -.01 | -.10 | -.03 | -.04 | -.12 | -.04 | -.12 | -.05 | -- | -- | -- |
| 17. Satiety Responsiveness | .02 | -.05 | -.08 | .12 | .14* | -.04 | -.08 | .03 | -.09 | -.07 | -.06 | -.10 | .04 | -.01 | -.12 | .16* | -- | -- |
| 18. Food Responsiveness | -.03 | -.10 | .02 | .03 | .14* | -.13 | .06 | .12 | .07 | .05 | .07 | .07 | .11 | .01 | -.20** | .16* | .04 | -- |
| 19. Enjoyment of Food | -.12 | -.13 | -.12 | -.09 | -.11 | -.02 | -.05 | .03 | .00 | -.09 | -.05 | -.01 | .10 | .05 | .11 | -.33*** | -.26*** | -.04 |

*Notes:* mYFAS = Modified Yale Food Addiction Scale, PFS = Power of Food Scale, MCP = Multiple Choice Procedure, RVFQ = Reinforcing Value of Food Questionnaire, UPF = Ultra-Processed Food, BMI = Body Mass Index, GWG = Gestational Weight Gain. ^a^Dummy coded (0 = Minority Race including Black, Asian, Hispanic or Latino, 1 = Non-Hispanic White). ^b^Log-transformed values. ^c^Reverse-scored value. ^d^Dummy coded (0 = Inadequate or adequate, 1 = Excessive). **p* < .05, ***p* < .01, ****p* < .001
